# Disharmony between wake- and respiration-promoting activities: effects of modafinil on ventilatory control in rodents

**DOI:** 10.1186/s12931-016-0466-9

**Published:** 2016-11-14

**Authors:** Jiro Terada, Isato Fukushi, Kotaro Takeda, Yohei Hasebe, Mieczyslaw Pokorski, Koichiro Tatsumi, Yasumasa Okada

**Affiliations:** 1 Department of Respirology, Graduate School of Medicine, Chiba University, 1-8-1 Inohana, Chuo-ku, Chiba city, Chiba 260-8670 Japan; 2 Clinical Research Center, Murayama Medical Center, 2-37-1 Gakuen, Musashimurayama, Tokyo, 208-0011 Japan; 3 Department of Biomedical Engineering, Graduate School of Science and Engineering, Toyo University, 2100 Kujirai, Kawagoe, Saitama 350-8585 Japan; 4 Fujita Memorial Nanakuri Institute, Fujita Health University, 423 Oodori-cho, Tsu, Mie 514-1296 Japan; 5 Department of Pediatrics, School of Medicine, University of Yamanashi, 1110 Shimokato, Chuo, Yamanashi 409-3898 Japan; 6 Opole Medical School, 68 Katowicka Street, Opole, 45-060 Poland

**Keywords:** Breathing, Electroencephalogram, Hypercapnic ventilatory response, Hypoxic ventilatory response, Narcolepsy, Respiratory failure, Vigilance, Wake-promoting drug

## Abstract

**Background:**

Modafinil is a wake-promoting drug and has been widely used for daytime sleepiness in patients with narcolepsy and other sleep disorders. A recent case series reported that daily oral modafinil alleviated hypercapnic respiratory failure in patients with COPD. However, the precise action of modafinil on respiration such as hypercapnic and/or hypoxic ventilatory responses remains unclear. The aim of this study is to clarify the effect of modafinil on the ventilatory control.

**Methods:**

We investigated the hypothesis that modafinil enhances resting ventilation as well as the stimulatory ventilatory responses to hypercapnia and hypoxia. We addressed the issue by examining minute ventilation, respiratory rate and volume components using plethysmography, combined with a concurrent EEG monitoring of the level of wakefulness before and after administration of modafinil in two doses of 100 mg/kg and 200 mg/kg in unanesthetized mice. In addition, we monitored the effect of the lower dose of modafinil on mice locomotor activity in a freely moving condition by video-recording.

**Results:**

Wakefulness, locomotor activity and variability of the breathing pattern in tidal volume were promoted by both doses of modafinil. Neither dose of modafinil increased the absolute values of resting ventilation or promoted the ventilatory responses to hypercapnia and hypoxia. Rather, higher dose of modafinil slightly suppressed respiratory rate in room air condition.

**Conclusions:**

Modafinil is conducive to the state of wakefulness but does not augment resting ventilation or the hyperventilatory responses to chemical stimuli in unanesthetized rodents.

**Electronic supplementary material:**

The online version of this article (doi:10.1186/s12931-016-0466-9) contains supplementary material, which is available to authorized users.

## Background

Modafinil is a widely-used wake-promoting drug for treating somnolence in narcolepsy and residual sleepiness persisting in obstructive sleep apnea despite continuous positive airway pressure treatment [1–3]. The drug activates brain areas involved in the control of arousal state through dopaminergic, orexinergic [4, 5], and histaminergic [6] neurotransmitter pathways, and the sympathetic nervous system. Recent studies on modafinil, in addition to a reduction in daytime sleepiness, have also demonstrated a spate of neurological effects such as cognitive enhancement and antidepressant effects [7, 8]. These multifarious actions of modafinil led us to hypothesize that it could affect ventilation [3].

The process of setting the ventilatory drive originates in the brain stem and is entwined with the central neural pathways above outlined, although this is an area of limited understanding. Notably, however, ventilation and its stimulatory responses to hypercapnia and hypoxia assume a depressive vein in sleep; the condition which modafinil is expected to counteract. It was thus a reasonable assumption that modafinil would enhance ventilation and its responses. The assumption was strengthened by a recent report demonstrating that modafinil, given orally on a daily basis, alleviated hypercapnic respiratory failure in COPD patients (i.e., improving oxygenation, and lowering the arterial carbon dioxide level) without adverse effects [9]. This off-label use of modafinil opens a new door to regulate hypercapnic respiratory failure in noncompliance patients with non-invasive ventilation [10]. However, the precise action of modafinil on the respiratory control system has not been fully investigated.

Additionally, traditional wake-promoting agents such as methylxanthines [11], methylphenidate [12], and amphetamine [13] have been known to cause some positive influence on respiratory control. Among those, methylxanthines have been clinically used, and their mechanisms of action have been well analyzed. For example, caffeine is frequently used for the treatment of apnea in premature infants, and also enhances the ventilatory response to hypoxia [14]. Theophylline and aminophylline prevent hypoxic ventilatory depression via inhibition of adenosine [15, 16]. Methylphenidate is known to induce emergence from general anesthesia by increasing arousal and respiratory drive [12].

In this study we seek to determine the effects of modafinil on resting ventilation in room air and on the stimulatory hypercapnic/hypoxic ventilatory responses in unanesthetized spontaneously breathing mice. We monitored the arousing central effects of modafinil by recording electroencephalogram (EEG) and by video-recording locomotor activity. In addition, the effect of modafinil on blood gas content was checked in unanesthetized spontaneously breathing rats. In contrast to our presumption, the investigation failed to demonstrate any appreciable effect of modafinil on ventilatory regulation.

## Methods

### Animals

Nine conscious, spontaneously breathing, male C57BL/6 mice (age 16.5 ± 0.6 weeks, weight 26.9 ± 0.6 g) were used in the study of ventilatory measurement. Further, to analyze arterial blood gas, six conscious, spontaneously breathing, male Wistar rats (age 25.0 ± 0.1 weeks, weight 284 ± 7 g) were used. The mice and rats were housed in separate cages at 23–24 °C, 50–60% relative humidity, and 12/12 h light/dark cycle, and were fed with commercial chow and water ad libitum. All experiments were conducted in sleep-time for rodents (9 am–5 pm).

### Experimental preparation

#### Recording of EEG

To monitor the functional status of the forebrain in mice, EEG was recorded. Surgical procedures were described in our previous report [17]. Briefly, EEG electrodes were implanted on the skull under isoflurane anesthesia, followed by intraperitoneally injected pentobarbital. The skull was exposed and three miniature screws were inserted; two were over the frontal lobes 2.5 mm posterior to the bregma as recording electrodes, and one was along the midline 4.5 mm anterior to the bregma as a ground electrode. The mice were allowed to recover from surgery for at least 1 week until EEG recordings began. EEG signals were amplified (JB-101 J and AB-651 J, Nihon Kohden, Tokyo, Japan) and bandpass filtered in 0.08–100 Hz frequency range.

#### Recording of ventilation

Resting ventilation, and the hypoxic and hypercapnic ventilatory responses were measured in a whole body rodent plethysmograph (PLY 310, EMMS, Bordon, UK) consisting of the recording (volume 530 mL) and reference chambers, as previously described [17–20]. Briefly, the chambers were placed inside a transparent acrylic box (size 20 × 20 × 20 cm). Each mouse was placed in the pre-calibrated recording chamber. Chamber temperature was maintained constant at 25 °C throughout the experiment. The air in the recording chamber was suctioned with a constant flow generator. To calculate the respiratory flow, the pressure difference between the recording and reference chambers was measured with a differential pressure transducer (TPF100, EMMS), connected to an amplifier (AIU060, Information & Display Systems, Bordon, UK), and was bandpass filtered at 0.1–20 Hz. The signal was integrated to obtain tidal volume (V_T_ [μL/weight (gram)]) for each respiratory cycle, which was then averaged throughout the period of interest. Respiratory rate (RR [breaths/min]) was counted. Minute ventilation (${\overset{.}{\mathrm{V}}}_{\mathrm{E}}$ [mL/g/min]) was calculated as V_T_ × RR. To evaluate the effect of modafinil on variability of the breathing pattern, the coefficients of variation of V_T_ and total respiratory cycle (a reciprocal of RR) were calculated as described in a previous study [21]. The O_2_ concentration in the chamber was continuously monitored with an O_2_ analyzer incorporating a polarographic sensor (Respina IH 26, San-ei, Tokyo, Japan), and was adjusted by controlling the mixing of nitrogen gas and air flows blown into the acrylic box. The pressure and EEG signals, together with O_2_ concentration data were simultaneously digitalized at 400 Hz sampling with an A/D converter (PowerLab4/26, ADInstruments, Colorado Springs, CO) and stored in a PC with LabChart7 software (ADInstruments).

#### Experimental protocols for whole body plethysmography

**Fig. 1:**
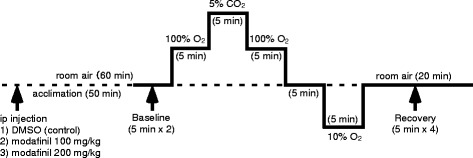
Scheme of the experimental protocol. See Methods for details of inspired gas mixture changes

Ventilation and its responses to sequential inspired gas changes before and after administration of a wake-promoting agent, modafinil were recorded in the tethering condition with EEG. The sequence of experimental steps is shown in Fig. 1. The hypercapnic (5% CO_2_) ventilatory response was analyzed in a hyperoxic condition to eliminate the hypoxic influence. The hypoxic ventilatory response was analyzed by loading 10% O_2_. Each gas mixture was maintained in the chamber for 5 min. Our experimental protocol of gas exchange (e.g., the pattern and length of gas exposures) was based on a previous report, which showed “time domain of ventilatory response” [22], and our previous studies, which adopted a protocol similar to the present study [17, 19, 20]. Because a concern was raised that resting ventilation or the hypoxic ventilatory response may be affected when hypoxic exposure was repeated. However, in our pilot study (*n* = 5, male C57BL/6 mice, age 15–17 weeks) repeating similar hypoxic exposure (see Fig. 1) with vehicle injection three times (DMSO trial 1–3) showed that ventilation was not confounded by the repeated hypoxic exposure, i.e., hypoxic ventilatory depression did not appear (see Additional file 1). Thus, the prior gas exposures should not have affected the ventilatory responses to the succeeding gas exposures in the present study. Modafinil (Sigma-Aldrich, St. Louis, MO) was dissolved with dimethyl sulfoxide (DMSO), and injected intraperitoneally in a volume of 0.5 ml/kg. After injection, 50 min was allowed for acclimation to a plethysmograph chamber and the following 10 min were taken as the baseline level of ventilation before the introduction of gas changes. Each exposure trial started in 60 min after injection of modafinil since previous research indicated peak response of modafinil in rodents especially for wakefulness is in 60–180 min after the injection [6, 23, 24]. This protocol was repeated, with intervals at least 10 min, in the following sequence:

1. DMSO alone (0.5 μL/g)
2. modafinil (lower dose, 100 mg/kg) in DMSO (0.5 μL/g)
3. modafinil (higher dose, 100 mg/kg, cumulative dose 200 mg/kg) in DMSO (0.5 μL/g).

The total dose of DMSO was 1.7 mg/kg, which does not affect respiratory function [20]. Although the doses of modafinil were certainly different from that used in humans, yet we used these two doses. This was because 50–300 mg/kg of modafinil adequately affects wakefulness and other physiological activities in rodents [4, 24, 25].

#### Blood gas analysis

Under anesthesia with isoflurane followed by pentobarbital injection (40 ~ 50 mg/kg, i.p.), a polyethylene catheter (Tombo No.9003 PFA, inner diameter 0.5 mm, outer diameter 1.0 mm, Nichias, Tokyo, Japan) was inserted into a unilateral femoral artery in rats to sample arterial blood. The catheter was filled with saline containing heparin, tunneled subcutaneously, and exteriorized at the dorsal midline between the bilateral scapulae. The exteriorized catheter tip was covered with a jacket to protect from biting by the rats. After catheterization, the rats were allowed to recover for at least 48 h. Then, DMSO and modafinil was injected in the following sequences same as the mice protocol: (1) DMSO alone, (2) modafinil (lower dose, 100 mg/kg) in DMSO, (3) modafinil (higher dose, 200 mg/kg) in DMSO. In blood gas analysis experiments, modafinil purchased from Alfresa Pharma (Osaka, Japan) was used. Sixty minutes after each injection, arterial blood (0.2 mL) was sampled, and arterial blood gas was analyzed (Blood Gas Analyzer ABL77, Radiometer, Copenhagen, Denmark). After each sampling of arterial blood, saline was intraarterially injected to maintain circulating blood volume constant.

### Video monitoring

To monitor the effect of modafinil on mice locomotor activity in a freely moving condition, behavior of mice in an ordinary cage was video-recorded before and after intraperitoneal administration of modafinil (100 mg/kg).

### Data analyses

In the measurement of ventilation, periods during which the mouse moved (e.g., sniffing, grooming, and licking) were excluded, but these periods were counted as locomotor activity (% period) in mice tethered with the EEG device. Data were given as mean ± standard error. The mean values of V_T_, RR, ${\overset{.}{\mathrm{V}}}_{\mathrm{E}}$, the coefficient of variations (V_T_ and total respiratory cycle) and locomotor activity were respectively submitted to a two factor within-subject analysis of variance (ANOVA), with three pharmacological conditions; vehicle and two doses of modafinil, and four air conditions; baseline room air, hypercapnia (5% CO_2_ in O_2_), hypoxia (10% O_2_ in N_2_), and recovery room air. Whenever necessary, a Greenhouse-Geisser adjustment was used to correct for violations of sphericity. We applied Bonferroni correction for the multiple comparisons in post-hoc test. Statistical significance was set at *P* < 0.05. The signal processing was performed using MATLAB 2015a (MathWorks, Natick, MA) and all statistical analyses were performed using SPSS 22.0 (IBM, NY).

## Results

Unceasing restlessness was observed in eight out of the nine mice approximately 30 min after administration of modafinil (100 mg/kg). One mouse, injected with the higher dose modafinil (200 mg/kg), died after the experimental protocol of hypoxic challenge was completed.

### Wakefulness and locomotor activity

**Fig. 2:**
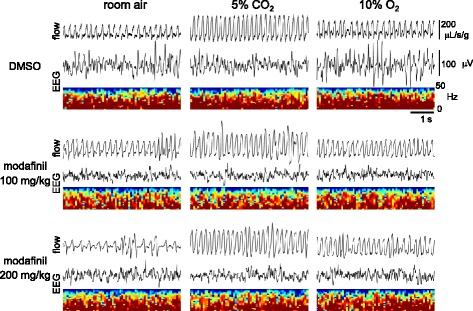
Representative traces of respiratory flow (plethysmographic signal, inspiration upward), EEG raw signal and its power spectrogram during room air, hypercapnic (5% CO_2_) and hypoxic (10% O_2_) conditions (see Fig. 1) after administration of DMSO and 100 and 200 mg/kg modafinil. EEG: electroencephalogram, DMSO: dimethyl sulfoxide

**Fig. 3:**
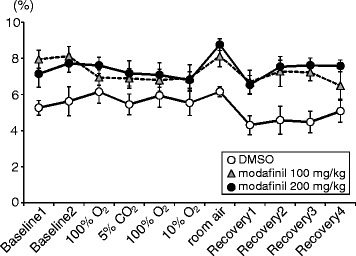
Locomotor activity during experimental protocols (see Fig. 1) in three pharmacological conditions: DMSO and 100 and 200 mg/kg modafinil. Five minutes were allocated in each gas change protocol (e.g., Baseline1, Baseline2, 10% O_2_). Significant differences among the three pharmacological conditions (*F*(2, 16) = 9.53, *P* < 0.01) and the four air conditions (*F*(3, 24) = 7.50, *ε*_GG_ = 0.57, *P* < 0.01) were revealed by the two-way repeated measures ANOVA. However, interaction was not significant (*F*(6, 48) = 1.20, *P* > 0.05). DMSO: dimethyl sulfoxide. Baseline: room air condition, Recovery: room air condition


A change in EEG signal to low amplitude and relatively high frequency was noted after injection of modafinil in the mice (Fig. 2). Locomotor activity was clearly promoted after injection of both low and high doses of modafinil (Fig. 3). The increase in locomotion was about the same for both doses of modafinil; the higher dose did not potentiate the effect. Nor were there any major changes in locomotion during consecutive gas mixtures swaps in the chamber, with a tendency for locomotion stimulation during hypoxia after modafinil. Video monitoring for the mice without EEG attachment in an ordinary cage showed that the movement of mouse injected with modafinil (right cage) was continuously activated compared with the control mouse (left cage) (see Additional file 2).

Additional file 2Different behaviors of mice in an ordinary cage observed after administration of modafinil 100 mg/kg (right cage) vs. DMSO (left cage). The video was recorded 45–50 min after administration of modafinil or DMSO. DMSO: dimethyl sulfoxide. (MP4 74823 kb)


### Respiration

**Fig. 4:**
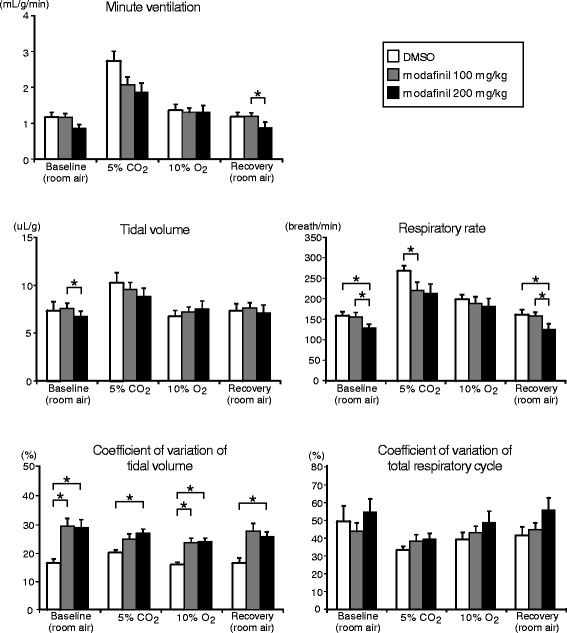
Minute ventilation (${\overset{.}{\mathrm{V}}}_{\mathrm{E}}$), tidal volume (V_T_), respiratory rate (RR), and coefficient of variations of V_T_ and total respiratory cycle in the three pharmacological conditions (DMSO and 100 and 200 mg/kg of the modafinil) in response to the four air conditions (baseline room air, 5% CO_2_, 10% O_2_, and recovery room air). Significant interactions between conditions were revealed by the two-way repeated measures ANOVA for ${\overset{.}{\mathrm{V}}}_{\mathrm{E}}$ (*F*(6, 48) = 5.54, *ε*_GG_ = 0.21, *P* < 0.05), V_T_ (*F*(6, 48) = 3.47, *ε*_GG_ = 0.39, *P* < 0.05), and the coefficient of variation of V_T_ (*F*(6, 48) = 2.96, *P* < 0.05). For RR, the interaction was marginally short of conventional level of statistical significance (*F*(6, 48) = 2.93, *ε*_GG_ = 0.38, *P* = 0.07). Post hoc Bonferroni test revealed some significant differences (*). For the coefficient of variation of total respiratory cycle, either the interaction between conditions or the main effect regarding pharmacological condition was not significant (*F*(6, 48) = 0.85, *P* = 0.85, and *F*(2, 16) = 2.62, *P* = 0.10, respectively). DMSO: dimethyl sulfoxide

The respiratory flow signal at rest changed to more irregular pattern along with promoted wakefulness after both doses injection of modafinil in the mice (see Fig. 2). Coefficient of variation of V_T_ (but not total respiratory cycle) was significantly enhanced in mice with modafinil, especially with higher dose of modafinil (Fig. 4). However, any absolute level of ventilation (i.e., either V_T_, RR, or ${\overset{.}{\mathrm{V}}}_{\mathrm{E}}$) was not promoted after modafinil injection, compared with the control injection of DMSO (Fig. 4). In contrast, baseline V_T,_ baseline and recovery RR in room air decreased after administration of higher doses of modafinil; recovery ${\overset{.}{\mathrm{V}}}_{\mathrm{E}}$ decreased with the higher dose of modafinil with a similar tendency of baseline ${\overset{.}{\mathrm{V}}}_{\mathrm{E}}$ (*P* = 0.051). Modafinil, in either dose, failed to appreciably affect ventilation during exposure to hypercapnia or hypoxia. The only exception was a slight but significant suppressive effect of modafinil on RR during the hypercapnic response observed at the lower dose of modafinil; with a similar tendency at the higher dose (Fig. 4). Note that tidal volume as shown in Fig. 4 was calculated as cumulative sum of respiratory flow in whole body plethysmography, but not solely determined by the magnitude of peak flow as shown in Fig. 2.

**Fig. 5:**
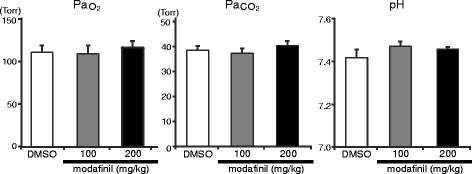
Blood gas analysis in spontaneous breathing rats at room air in the two pharmacological conditions: DMSO and 100 and 200 mg/kg of the modafinil. No significant differences among the three pharmacological conditions were observed. DMSO: dimethyl sulfoxide

Arterial blood gas values (Pa_O2_, Pa_CO2_ and pH) in six rats did not change after injection of modafinil, compared with control (Fig. 5).

## Discussion

The major finding of this study is that modafinil, a wake-promoting medicine, is not conducive to ventilation and its stimulatory responses to chemical stimuli. The study demonstrates, therefore, that wake-promoting cortical function, as assessed from the EEG and locomotion recordings in the present study, is not congruous with the central regulation of ventilation by the brain. Further, modafinil, showed some suppressive effect on respiratory rate. Yet this effect was rather mild, failing to cause untoward changes in blood gas content. These findings run against our working hypothesis in that, while confirming that modafinil promotes wakefulness, we failed to lend support for the notion of its being augmentative for ventilation and ventilatory reactivity in conscious rodents. This hypothesis was ideated on the premise that cortical function is a key controller of the brain stem respiratory network [26], and integration of hyperventilatory neural inputs: hypercapnic emanating from central medullary chemoreceptors [27, 28] and hypoxic emanating from carotid chemoreceptors [29]. The premise was grounded in the fact that wakefulness and locomotion augment ventilation and its reactivity [26] as opposed to natural sleep or anesthetic-induced sleep [30, 31]. Thus, the finding of the lack of ventilatory augmentative effect of modafinil at a time of apparent cortical activation was rather unexpected. Our findings suggest the biological plausibility that cortical regulation of wakefulness and respiration proceeds from dichotomy to a possibly merge in lower brain areas where it loses selectivity. Modafinil would then act predominantly on the brain cortex.

The interpretation of the present findings that modafinil, which acts in narcoleptic patients by enhancement of cortical excitability [32, 33], does not promote but might rather suppress ventilation is backed by previous studies on the suprapontine mechanisms in the regulation of respiration in decerebrated and decorticated cats conducted in the 1960–1980s [34, 35]. In those reports, resting ventilation and hypoxic ventilatory response were enhanced in decorticated cats, suggesting that the cortex has a ventilatory suppressive property. Thus, we surmise that modafinil enhance the variability of the breathing pattern in V_T_ with promoted wakefulness as mentioned in previous literature [36], but does not facilitate absolute level of ventilation due to its predominantly cortical site of action rather than the brain stem respiratory network that is affected by other traditional wake-promoting drugs [11, 12, 14]. The exact neuronal mechanism underlying the action of modafinil was not the issue of the present study as it requires alternative study designs. The enhanced wakefulness and locomotor activity revealed in the present study after both low and high doses of modafinil has been demonstrated in other studies on the subject [23, 24, 37]. However, the finding of a ventilatory suppressive effect of modafinil, particularly on the hypercapnic response, albeit slight, was fairly unexpected in view of a recent report demonstrating that daily oral modafinil treatment alleviates hypercapnic respiratory failure in COPD patients [10]. The discrepancy might be explicable by different methodological approaches: 1) human vs. rodents, 2) human vs. experimental animal dose (100–200 mg/body vs. 100–200 mg/kg), 3) dosage regimen (daily for 2 weeks vs. one time only), and 4) condition (hypercapnic respiratory failure patients vs. healthy normocapnic animals).

This study has some limitations to be considered. Firstly, one time application of modafinil may not be sufficient for the evaluation of a change in ventilatory control as judged from a report of gradual alleviation of respiratory failure in COPD patients during chronic daily dosing of modafinil [9]. Secondly, the present study was conducted in healthy animals. Deteriorated ventilation in an animal model of respiratory failure could be more affected by modafinil. Modafinil displays cortical enhancing activity in narcoleptic, but not in healthy subjects [27]. Thirdly, we only analyzed short-term ventilatory responses to hypercapnia and hypoxia (5 min recording per each protocol, see Fig. 1). The result of longer term observation might be different, particularly that hypercapnia and hypoxia per se promote wakefulness in short-term; on which background the action of modafinil might be subdued. Fourthly, we used animals of different species, i.e., mice in the measurement of ventilation and rats in blood gas analyses. The findings in both measurements agreed and indicated the absence of ventilatory stimulatory action of modafinil. However, higher dose of modafinil slightly suppressed respiratory rate in room air condition in mice but did not affect blood gas in rats. This discrepancy may be explained by species difference and by relative weakness of ventilatory suppressive action of modafinil, as lower dose of modafinil did not affect either respiratory rate, tidal volume, or blood gas. Lastly, concerns may be raised that resting ventilation or the hypoxic ventilatory response may be confounded even without modafinil when hypoxic exposure was repeated. It may occur due to centrally- or muscle fatigue-mediated hypoxic ventilatory depression. However, our pilot study (see Additional file 1) showed that it does not occur at least in our gas exposure protocol. Nonetheless, the conclusion that modafinil does not promote respiration remains robust regardless of the experimental limitations.

The advantage of the present study is that it shows that modafinil is highly selective in its wake-promoting activity and is not concurrently conducive to ventilation and its responsiveness to chemical stimuli of hypercapnia and hypoxia. These findings reinforce clinical safety of modafinil for patients with narcolepsy or other daytime sleepiness, without adverse increase in metabolic rate that could lead to increased ventilation. The disadvantage, however, is that we were unable to unravel another facet of modafinil action, intuitively presumed, i.e., ventilatory facilitation, which could be of clinical interest in view of the paucity of true and safe ventilatory stimulants.

## Conclusion

We conclude that modafinil promotes wakefulness but not ventilation or its responsiveness to chemical stimuli in unanesthetized rodents.

## Additional files

Additional file 1: A pilot study for change in minute ventilation, tidal volume, and respiratory rate after repeated series of hypoxic exposure. Ventilatory parameters (minute ventilation, tidal volume, and respiratory rate) in the resting room air condition and their responses to hypoxia (10% O_2_) did not change while hypoxic exposure with vehicle injection (DMSO trial 1–3: DMSO 0.5 μL/g each) was repeated three times. DMSO, dimethyl sulfoxide. (PDF 100 kb)

## Abbreviations

DMSO: Dimethyl sulfoxide
EEG: Electroencephalogram
RR: Respiratory rate
$$ {\overset{.}{\mathrm{V}}}_{\mathrm{E}} $$: Minute ventilation
V_T_: Tidal volume
