# Supplementary figures and images for: Disharmony between wake- and respiration-promoting activities: effects of modafinil on ventilatory control in rodents
Source: Respir Res. 2016 Nov 14;17:148. doi: 10.1186/s12931-016-0466-9 (PMC5109771; doi:10.1186/s12931-016-0466-9)

## Minute ventilation

(mL/g/min)

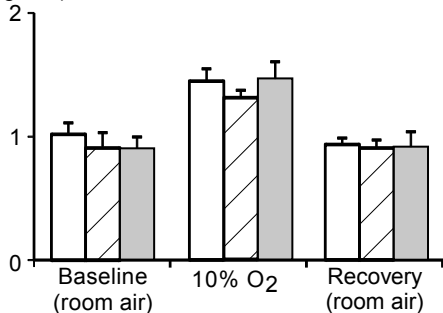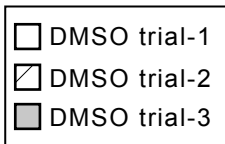

## Tidal volume

(uL/g)

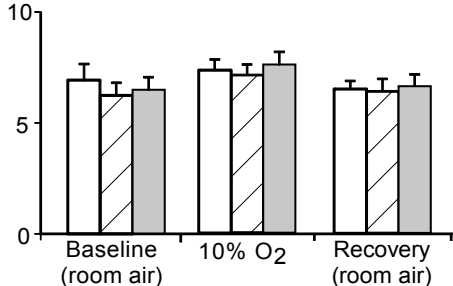

## Respiratory rate

(breath/min)

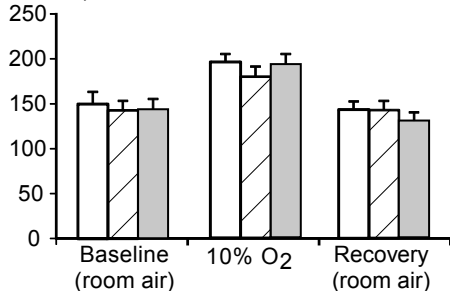

Supplement: Additional file 1: — A pilot study for change in minute ventilation, tidal volume, and respiratory rate after repeated series of hypoxic exposure. Ventilatory parameters (minute ventilation, tidal volume, and respiratory rate) in the resting room air condition and their responses to hypoxia (10% O2) did not change while hypoxic exposure with vehicle injection (DMSO trial 1–3: DMSO 0.5 μL/g each) was repeated three times. DMSO, dimethyl sulfoxide. (PDF 100 kb) [file 12931_2016_466_MOESM1_ESM.pdf]
